# Association Between Hospital Adoption of an Emergency Department Treatment Pathway for Opioid Use Disorder and Patient Initiation of Buprenorphine After Discharge

**DOI:** 10.1001/jamahealthforum.2023.0245

**Published:** 2023-03-24

**Authors:** Keisha T. Solomon, Jason O’Connor, Jason B. Gibbons, Austin S. Kilaru, Kenneth A. Feder, Lingshu Xue, Brendan Saloner, Elizabeth A. Stuart, Evan S. Cole, Eric Hulsey, Zachary Meisel, Esita Patel, Julie M. Donohue

**Affiliations:** 1 Department of Economics, Howard University, Washington, DC; 2 Department of Health Policy and Management, Johns Hopkins University Bloomberg School of Public Health, Baltimore, Maryland; 3 Department of Health Policy and Management, University of Pittsburgh School of Public Health, Pittsburgh, Pennsylvania; 4 University of Pennsylvania, Philadelphia, Pennsylvania; 5 Department of Mental Health, Johns Hopkins University Bloomberg School of Public Health, Baltimore, Maryland; 6 Vital Strategies, New York, New York; 7 Advanced Practice Outcomes and Analytics, The Center for Advanced Practice, Atrium Health Carolinas Medical Center, Charlotte, North Carolina

## Abstract

**Importance:**

Emergency department (ED)–based initiation of buprenorphine has been shown to increase engagement in outpatient treatment and reduce the risk of subsequent opioid overdose; however, rates of buprenorphine treatment in the ED and follow-up care for opioid use disorder (OUD) remain low in the US. The Opioid Hospital Quality Improvement Program (O-HQIP), a statewide financial incentive program designed to increase engagement in OUD treatment for Medicaid-enrolled patients who have ED encounters, has the potential to increase ED-initiated buprenorphine treatment.

**Objective:**

To evaluate the association between hospitals attesting to an ED buprenorphine treatment O-HQIP pathway and patients’ subsequent initiation of buprenorphine treatment.

**Design, Setting, and Participants:**

This cohort study included Pennsylvania patients aged 18 to 64 years with continuous Medicaid enrollment 6 months before their OUD ED encounter and at least 30 days after discharge between January 1, 2016, and December 31, 2020. Patients with a claim for medication for OUD 6 months before their index encounter were excluded.

**Exposures:**

Hospital implementation of an ED buprenorphine treatment O-HQIP pathway.

**Main Outcomes and Measures:**

The main outcome was patients’ receipt of buprenorphine within 30 days of their index OUD ED visit. Between August 2021 and January 2023, data were analyzed using a difference-in-differences method to evaluate the association between hospitals’ O-HQIP attestation status and patients’ treatment with buprenorphine after ED discharge.

**Results:**

The analysis included 17 428 Medicaid-enrolled patients (female, 43.4%; male, 56.6%; mean [SD] age, 37.4 [10.8] years; Black, 17.5%; Hispanic, 7.9%; White, 71.6%; other race or ethnicity, 3.0%) with OUD seen at O-HQIP–attesting or non–O-HQIP–attesting hospital EDs. The rate of prescription fills for buprenorphine within 30 days of an OUD ED discharge in the O-HQIP attestation hospitals before the O-HQIP intervention was 5%. The O-HQIP attestation was associated with a statistically significant increase (2.6 percentage points) in prescription fills for buprenorphine within 30 days of an OUD ED discharge (β, 0.026; 95% CI, 0.005-0.047).

**Conclusions and Relevance:**

In this cohort study, the O-HQIP was associated with an increased initiation of buprenorphine in patients with OUD presenting to the ED. These findings suggest that statewide incentive programs may effectively improve outcomes for patients with OUD.

## Key Points

**Question:**

What is the association between hospitals attesting to an emergency department buprenorphine treatment Opioid Hospital Quality Improvement Program (O-HQIP) pathway and patients’ subsequent initiation of buprenorphine treatment?

**Findings:**

In this cohort study using difference-in-differences analyses of 17 428 Medicaid-enrolled adults with an opioid use disorder emergency department encounter, the buprenorphine treatment O-HQIP pathway was associated with significantly increased prescription fills for buprenorphine within 30 days of discharge.

**Meaning:**

This study’s findings suggest that the O-HQIP may be a new policy approach to expanding access to evidence-based treatment for opioid use disorder.

## Teaser

This cohort study evaluates the association between hospitals attesting to an opioid use disorder (OUD) emergency department (ED) treatment pathway and patients’ initiation of buprenorphine treatment.

## Introduction

Emergency departments (EDs) have become the front line of the opioid overdose epidemic, which in 2021 claimed the lives of approximately 80 000 Americans.^1^ In addition to stabilizing patients following an opioid overdose, EDs are crucial touchpoints for engaging patients in treatment for opioid use disorder (OUD). Recent efforts have sought to expand access to treatment with buprenorphine during an ED encounter and link patients to outpatient follow-up services (also known as a warm handoff).^2,3,4,5^ Emergency department–based initiation of buprenorphine, which is an effective medication treatment for OUD, has been shown to increase engagement in outpatient treatment and reduce the risk of subsequent opioid overdose.^6,7^ However, rates of buprenorphine treatment in the ED and follow-up care for OUD remain low in the US.^2^ Policy makers and payers can support the adoption of ED-based buprenorphine and warm-handoff programs in hospitals through financial incentives, among other policy approaches.

Medicaid is the largest payer for OUD treatment in the US. Medicaid covered nearly 40% of American adults with OUD in 2017.^8^ In response to high opioid overdose death rates, the Pennsylvania Department of Human Services (DHS) implemented the Opioid Hospital Quality Improvement Program (O-HQIP) in 2019.^9^ The O-HQIP is the first statewide financial incentive program designed to increase engagement in OUD treatment, including buprenorphine treatment, for Medicaid-enrolled patients who have ED encounters.^10,11^

To increase engagement in OUD treatment within 7 days of an OUD ED encounter, the O-HQIP was implemented in 2 phases. In its first phase, the O-HQIP provided payments to hospitals that agreed to implement 1 or more of 4 clinical pathways: (1) ED initiation of buprenorphine, (2) warm handoff to community treatment resources, (3) dedicated protocols for pregnant people with OUD, and (4) hospitalization for induction of medication for OUD (MOUD). The DHS announced the O-HQIP to hospitals in 2018. Hospitals opting to participate in the program’s first phase were then required to submit clearly defined pathways by September 2018, and pathways were verified as operational in January 2019.^12^ Participating hospitals received payments in July 2019 that varied from $37 000 to $193 000, depending on the chosen pathway^11^; the magnitude of these payments was independent of other factors, including hospital size and location. No penalties were associated with the O-HQIP program.^10^ Approximately 75% of Pennsylvania hospitals were determined to have attested to at least 1 O-HQIP pathway.^13,14^

In the O-HQIP’s second phase, which started in 2020, all Pennsylvania hospitals received payments based on annual performance metrics based on improvements in the proportion of Medicaid enrollees receiving substance use treatment within 7 days of an ED discharge for OUD.^11^ Starting in August 2019, all Pennsylvania hospitals had the option of participating in a 2-year statewide quality improvement collaborative, the Hospital and Healthsystem Association of Pennsylvania Opioid Learning Action Network (OLAN), in which hospitals jointly worked to improve and implement effective OUD health care delivery strategies.^15^

A qualitative study of representatives from hospitals participating in the O-HQIP in 2019 suggested that this initiative motivated hospitals to improve OUD treatment, but hospitals continued to face challenges in implementing buprenorphine induction in the ED.^10^ Although the Pennsylvania DHS collected data before and after program implementation, this study is the first to evaluate how ED-initiated buprenorphine treatment changed when hospitals attested to O-HQIP participation relative to hospitals that did not. In this study, we analyzed claims for a cohort of Pennsylvania Medicaid enrollees treated in an ED for OUD between 2017 and 2020 to estimate the association between hospitals attesting to an ED buprenorphine treatment pathway and patients’ subsequent initiation of buprenorphine treatment.

## Methods

### Study Population

In this cohort study, we obtained data from the Pennsylvania DHS on inpatient, outpatient, and pharmacy claims on a census of enrollees in Pennsylvania Medicaid from January 1, 2016, to December 31, 2020. We used Medicaid enrollment files to obtain enrollee demographic characteristics, enrollment duration, and eligibility categories.

This project was reviewed and considered exempt by the institutional review boards at the Johns Hopkins Bloomberg School of Public Health and the University of Pittsburgh; thus, the study did not require informed consent because patient data were deidentified. The study followed the Strengthening the Reporting of Observational Studies in Epidemiology (STROBE) reporting guideline.

Our initial study cohort consisted of patients aged 18 to 64 years who (1) visited an ED for an opioid-related cause between January 1, 2017, and December 31, 2020, and (2) were enrolled continuously in Medicaid during the 6 months before their ED encounter and for at least 30 days after discharge. We used procedure and revenue codes in the Medicaid outpatient claims files to identify patients’ ED encounters. To classify an ED encounter as opioid related, we used the presence of 1 or more *International Classification of Diseases, Tenth Revision* F11 codes (ie, diagnoses for opioid dependence, withdrawal, abuse, overdose) or T40 codes (ie, diagnoses for opioid overdose) during the ED stay (eTable 1 in Supplement 1).

We considered only the first OUD ED encounter in our analysis (hereafter, index encounter) for patients who had multiple encounters between 2017 and 2020. We excluded patients with an index encounter in the first 6 months of 2017 who had an ED encounter 180 days before their index encounter and patients who were eligible for Medicare but had an incomplete claims capture. We also excluded patients who had a claim for MOUD during the 6 months before their index encounter, because our goal was to evaluate MOUD initiation among recently treatment-naive patients rather than a continuation of previous treatment. We also excluded a small number of enrollees (approximately 1%) with missing sociodemographic characteristics used in our analyses (Figure).

**Figure.**
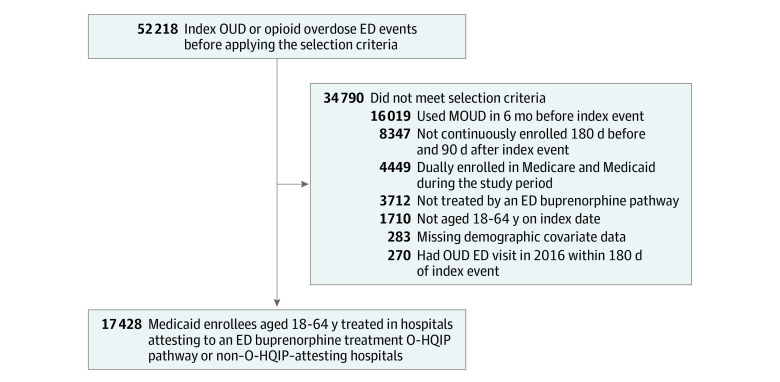
Selection of the Study Population Selection criteria were applied to Pennsylvania Medicaid enrollees who visited an emergency department (ED) for an opioid-related cause between January 1, 2017, and December 31, 2020. MOUD indicates medication for opioid use disorder; O-HQIP, Opioid Hospital Quality Improvement Program; and OUD, opioid use disorder.

We evaluated the association of hospitals’ attestation to ED initiation of buprenorphine (ie, O-HQIP pathway 1) because of the strength of prior evidence for the effectiveness of buprenorphine in the ED setting^16^ and the potential for that pathway attestation for helping hospitals to overcome the challenges of implementing buprenorphine in the ED setting.^10^ Furthermore, the majority of O-HQIP–participating hospitals attested to pathway 1.^17^ Therefore, our analysis compared patients who received care at hospitals participating in O-HQIP pathway 1 with hospitals that did not participate in any O-HQIP pathway. Thus, we excluded patients treated at hospitals that only participated in O-HQIP pathways 2, 3, or 4.

### Variable Measurement

#### Intervention

Our key independent variable is an indicator for whether a patient’s index OUD ED visit occurred during or after January 2019 (the start date for the O-HQIP) at a hospital that attested to O-HQIP pathway 1, henceforth referred to as an O-HQIP–attesting hospital. The preintervention period is 2017-2018, and the postintervention period is 2019-2020. The control group included patients seen at hospitals that did not attest to any O-HQIP pathway (ie, non–O-HQIP–attesting hospitals).

#### Outcomes

To analyze patient treatment with buprenorphine following discharge from the ED at a Pennsylvania hospital, we created an indicator that captured a buprenorphine claim for OUD within 30 days of their index OUD ED visit. To maintain compliance with Centers for Medicare & Medicaid Services regulations prohibiting data reporting with cell sizes of fewer than 11 individuals, we estimated 30-day instead of 7-day outcomes because some subgroups with 7-day outcomes were too small to report. We identified buprenorphine prescription claims from Medicaid pharmacy claims and buprenorphine administrations from Medicaid outpatient and/or professional claims (eTable 1 in Supplement 1).

#### Covariates

We included sociodemographic characteristics measured in Medicaid enrollment files, including sex (female and male), age at index visit, race and ethnicity (Black, Hispanic, White, and other [Alaska Native, American Indian, Asian, Native Hawaiian, and Pacific Islander]), Medicaid eligibility group (pregnant women, children, individuals with disabilities or chronic illness, adults without disabilities, and Medicaid expansion adults), managed care organization region of residence (Lehigh Capital, New East, New West, Southeast, and Southwest), urban location (metropolitan areas), and rural location (micropolitan, small town, and other nonmetropolitan areas). To classify race and ethnicity, we used Medicaid enrollment files from the Pennsylvania DHS that included demographic characteristics of Medicaid enrollees. Race and ethnicity was measured by beneficiary self-report at the time of enrollment. We controlled for race and ethnicity because of racial and ethnic disparities in the use of MOUD.^18,19,20,21^

We used diagnoses from Medicaid inpatient, outpatient, and professional claims files to identify clinical characteristics associated with engagement in treatment or the severity of OUD, including mental health diagnoses (anxiety disorder, mood disorder, schizophrenia and other psychosis, and posttraumatic stress disorder), hepatitis C virus, HIV, abscess, osteomyelitis, endocarditis, and soft skin tissue infection. We also adjusted for treatment use 6 months before the patient’s index ED encounter as follows: frequency of inpatient ED admissions, outpatient visits, opioid prescription fills, benzodiazepine prescription fills, and unique prescribers of opioids.

### Statistical Analysis

Data were analyzed between August 2021 and January 2023. We first described the sociodemographic and clinical characteristics of the study population by O-HQIP attestation status for the pre- and postintervention periods. For the primary analysis, we used a difference-in-differences (DD) design to evaluate the association between a hospital’s attestation to the O-HQIP pathway 1 and a patient’s treatment with buprenorphine following discharge from the ED. Specifically, we fit a linear probability model for whether the patient received a buprenorphine prescription within 30 days of their index OUD ED visit. The key explanatory variable is an indicator of whether a patient was treated at an O-HQIP–attesting hospital after January 1, 2019. Models also included hospital-level fixed effects to control for time-invariant hospital unobserved effects and dummy variables for the calendar period (before vs after January 1, 2019). In adjusted models, we controlled for the patient sociodemographic and clinical characteristics to account for potential differences in patient characteristics between those whose index OUD ED stay was at an O-HQIP– vs a non–O-HQIP–attesting hospital.

In addition to the overall association between O-HQIP attestation and treatment outcomes, we evaluated the intervention’s year-specific associations because of the potential delayed impact of the intervention on patients’ use of buprenorphine. In this model, we replaced our key explanatory variable with 2 indicator variables: 1 for patients visiting O-HQIP–attesting hospitals in 2019 and 1 for visits occurring in 2020. Both indicators were compared with the preintervention period.

For all specifications, we estimated unadjusted and adjusted regression models. Standard errors were clustered at the hospital level to account for correlated patient outcomes within hospitals. Regression coefficients are to be interpreted as percentage-point differences. For each coefficient, we estimated 95% Wald CIs. All analyses were performed using R, version 4.1.1 statistical software (R Foundation for Statistical Computing).

We conducted 4 additional analyses. First, we estimated models with additional controls to reduce the likelihood that other unobserved factors led to biased DD estimates. In particular, we included the hospital’s OLAN participation status as a covariate. In a separate model, we accounted for a patient’s history of buprenorphine treatment as an indicator variable for whether the patient received MOUD 7 to 12 months before their index visit.

Second, because O-HQIP–attesting hospitals may differ from those not attesting by other hospital-level and patient composition characteristics associated with patients’ probability of initiating buprenorphine treatment, we created a subsample based on attestation probabilities. We used logistic regression to evaluate the association of O-HQIP attestation with measured hospital-level and patient composition characteristics (variables are listed in eTable 2 in Supplement 1) to obtain the probabilities. This matching process substantially reduced the sample size. However, patients in the matched sample had similar characteristics to those in the full sample (eTable 3 in Supplement 1).

Third, to explore the similarity of the O-HQIP–attesting and non–O-HQIP–attesting hospitals during the preintervention period, we created an exploratory plot showing the monthly prevalence of 30-day buprenorphine treatment for patients who visited attesting and nonattesting hospitals (eFigure in Supplement 1). However, the data on monthly 30-day buprenorphine treatment were noisy, which prevented a comparative interrupted time-series analysis and made visual inspection of parallel trends difficult. Therefore, we conducted an additional analysis to explore the assumption of parallel counterfactual trends. In particular, we repeated the main analyses using a false treatment implementation date of April 1, 2018 (ie, 8 months before the actual program implementation date), and only encounters before O-HQIP implementation. A significant intervention effect could indicate that intervention effects observed in the primary analysis are attributable to preexisting divergence over time in the incidence of the outcome between attesting and nonattesting hospitals.

Fourth, we explored the spillover association of the O-HQIP program with other measures of substance use disorder (SUD) treatment. We evaluated patient treatment with naltrexone, methadone, and non-MOUD (SUD treatment service that did not include medication, eg, residential treatment, counseling) separately, all within 30 days of an OUD ED encounter. Because the performance metrics of O-HQIP’s second phase focused on patients’ receipt of SUD treatment within 7 days, we also evaluated an outcome that captured a claim for any SUD treatment (non-MOUD, buprenorphine, methadone, or naltrexone) within 7 days of a patient’s index OUD ED visit.

## Results

Our main study sample included 17 428 patients with OUD (female, 43.4%; male, 56.6%; mean [SD] age, 37.4 (10.8) years; Black, 17.5%; Hispanic, 7.9%; White, 71.6%; other race or ethnicity, 3.0%) seen at O-HQIP–attesting hospitals (n = 14 585) or non–O-HQIP–attesting hospitals (n = 2843) whose index ED visits met all study criteria (Figure). The baseline rate for patients prescribed buprenorphine within 30 days of their ED visit for an opioid overdose was similar between O-HQIP–attesting and non–O-HQIP–attesting hospitals (5.0% vs 5.7%). Overall, patients who visited O-HQIP–attesting and non–O-HQIP–attesting hospitals had similar demographic and clinical characteristics during the preintervention period (2017-2018), except that patients treated at O-HQIP–attesting hospitals were more likely to be Black or Hispanic (16.8% vs 7.1% and 7.9% vs 5.2%, respectively) (Table 1).

**Table 1.** Characteristics of the Study Population^a^

Characteristic	No. (%)			
	O-HQIP–attesting hospitals		Non–O-HQIP–attesting hospitals	
	2017-2018 (n = 9120)	2019-2020 (n = 5465)	2017-2018 (n = 1899)	2019-2020 (n = 944)
Treatment with buprenorphine within 30 d of index visit	456 (5.0)	447 (8.2)	109 (5.7)	59 (6.2)
**Patient characteristics**				
Age at index visit, mean (SD), y	37.25 (10.64)	38.31 (11.09)	35.43 (10.14)	37.47 (10.87)
Sex				
Female	3958 (43.3)	2292 (41.9)	894 (47.1)	426 (45.1)
Male	5162 (56.6)	3173 (58.1)	1005 (52.9)	518 (54.9)
Race and ethnicity				
Black	1529 (16.8)	1275 (23.3)	134 (7.1)	107 (11.3)
Hispanic	717 (7.9)	504 (9.2)	98 (5.2)	59 (6.2)
White	6628 (72.7)	3488 (63.8)	1623 (85.5)	747 (79.1)
Other^b^	246 (2.7)	198 (3.6)	44 (2.3)	31 (3.3)
Eligibility category				
Children	197 (2.2)	119 (2.2)	58 (3.1)	27 (2.9)
Disability or chronic illness	1598 (17.5)	1007 (18.4)	260 (13.7)	167 (17.7)
Adults without disabilities	1277 (14.0)	784 (14.3)	262 (13.8)	147 (15.6)
Medicaid expansion adults	5864 (64.3)	3484 (63.8)	1264 (66.6)	594 (62.9)
Managed care organization region				
Lehigh Capital	1474 (16.2)	918 (16.8)	264 (13.9)	125 (13.2)
New East	874 (9.6)	516 (9.4)	929 (48.9)	430 (45.6)
New West	636 (7.0)	320 (5.9)	155 (8.2)	109 (11.5)
Southeast	3013 (33.0)	2074 (38.0)	420 (22.1)	218 (23.1)
Southwest	3123 (34.2)	1637 (30.0)	131 (6.9)	62 (6.6)
Address in rural area	924 (10.1)	493 (9.0)	317 (16.7)	218 (23.1)
Comorbidities				
Hepatitis C virus	1313 (14.4)	601 (11.0)	250 (13.2)	98 (10.4)
Anxiety disorder	3714 (40.7)	2192 (40.1)	769 (40.5)	403 (42.7)
Mood disorder	4191 (46.0)	2307 (42.2)	854 (45.0)	411 (43.5)
Schizophrenia or other psychotic disorder	812 (8.9)	571 (10.4)	173 (9.1)	81 (8.6)
Posttraumatic stress disorder	658 (7.2)	510 (9.3)	119 (6.3)	99 (10.5)
Skin and soft tissue infections	1305 (14.3)	620 (11.3)	257 (13.5)	119 (12.6)
**Hospital characteristics, mean (SD)**				
No. of outpatient visits	3.61 (5.74)	3.42 (4.66)	3.43 (4.42)	3.49 (4.42)
No. of inpatient admissions	0.43 (1.24)	0.41 (1.24)	0.34 (0.81)	0.34 (0.98)
No. of opioid prescriptions fills	0.98 (2.57)	0.42 (1.63)	0.88 (2.64)	0.47 (1.71)
No. of benzodiazepine prescriptions	0.83 (2.12)	0.52 (1.75)	0.85 (2.12)	0.65 (1.96)
Elixhauser comorbidity index score at the index ED visit	2.11 (2.51)	2.12 (2.59)	1.94 (2.36)	1.99 (2.53)
No. of unique prescribers of opioids	0.35 (0.78)	0.19 (0.57)	0.30 (0.72)	0.21 (0.59)

Abbreviations: ED, emergency department; O-HQIP, Opioid Hospital Quality Improvement Program.

^a^ The number of outpatient visits, inpatient admissions, opioid prescriptions, benzodiazepine prescription fills, and unique prescribers of opioids were measured 6 months prior to the patient’s index ED visit. For confidentiality purposes and adherence to Centers for Medicare & Medicaid Services regulations, we do not report statistics when a small number of individuals (ie, <11) had the relevant characteristic.

^b^ Other race and ethnicity includes Alaska Native, American Indian, Asian, Native Hawaiian, and Pacific Islander.

Table 2 reports DD regression results for the overall and year-specific associations between O-HQIP attestation and receipt of buprenorphine. Estimates from unadjusted analyses (model A1) were similar to estimates from the adjusted analyses (model A2). eTable 4 in Supplement 1 shows covariate estimates from adjusted analyses. Controlling for patient characteristics and calendar period, DD estimates show that O-HQIP attestation was associated with a 2.6 percentage-point increase in patients obtaining buprenorphine within 30 days of discharge (β, 0.026; 95% CI, 0.005-0.047). Our secondary analysis that evaluated the year-specific effects of O-HQIP attestation indicates that the overall pooled estimate was driven by associations in the second year of O-HQIP implementation. In the adjusted model, we found modest increases in 30-day buprenorphine treatment rates associated with O-HQIP attestation, with gains of 3.7 percentage points (β, 0.037; 95% CI, 0.015-0.059) in 2020 and 1.6 percentage points (β, 0.016; 95% CI, −0.012 to 0.045) in 2019 compared with the preintervention period. Results from models that accounted for the hospital’s OLAN participation or patient’s buprenorphine treatment history were similar to our main DD estimates (models B1, B2, C1, and C2) (Table 2).

**Table 2.** Association of the Opioid Hospital Quality Improvement Program (O-HQIP) With Patient Treatment With Buprenorphine Within 30 Days of Index Emergency Department Visit, Full Sample (17 428 Observations)^a^

Model	β (95% CI)		
	Overall before to after, O-HQIP vs no O-HQIP implementation	Year-specific indicators for post–O-HQIP period	
		2019	2020
**A: Main analysis**			
A1: Unadjusted models			
Buprenorphine within 30 d	0.025 (0.005 to 0.046)^b^	0.016 (−0.013 to 0.045)	0.038 (0.015 to 0.060)^b^
A2: Adjusted models			
Buprenorphine within 30 d	0.026 (0.005 to 0.047)^b^	0.016 (−0.012 to 0.045)	0.037 (0.015 to 0.059)^b^
**B: Analysis with OLAN as an additional covariate**			
B1: Unadjusted models			
Buprenorphine within 30 d	0.025 (0.004 to 0.047)^b^	0.016 (−0.012 to 0.044)	0.035 (0.008 to 0.063)^b^
B2: Adjusted models			
Buprenorphine within 30 d	0.025 (0.003 to 0.046)^b^	0.015 (−0.014 to 0.043)	0.036 (0.008 to 0.063)^b^
**C: Analysis with prior buprenorphine treatment as an additional covariate**			
C1: Unadjusted models			
Buprenorphine within 30 d	0.025 (0.005 to 0.045)^b^	0.015 (−0.011 to 0.044)	0.037 (0.015 to 0.060)^b^
C2: Adjusted models			
Buprenorphine within 30 d	0.026 (0.005 to 0.046)^b^	0.016 (−0.013 to 0.044)	0.038 (0.016 to 0.061)^b^

Abbreviation: OLAN, Opioid Learning Action Network.

^a^ In the model that evaluates the overall association, the coefficient of interest (O-HQIP attesting) is an indicator for whether a patient’s index opioid use disorder emergency department visit occurred during or after January 2019 (the start date for the O-HQIP) at O-HQIP–attesting hospitals. In the model that evaluates the year-specific effects, the 2 coefficients of interest are 2 indicator variables: 1 for patients visiting attesting hospitals in 2019 and 1 for 2020. All analyses used linear regression at the patient level and included an indicator variable for whether the patient was treated at an O-HQIP–attesting hospital after January 1, 2019. All models also included hospital-level fixed effects to control for time-invariant hospital unobserved effects. The sample sizes of the overall and year-specific models are equal (ie, 17 428). Clustering was performed at the hospital level.

^b^ Statistically different from 0 at the 5% level. Adjusted regression models also controlled for patient characteristics, including age (at the time of index opioid use disorder visit), sex, race and ethnicity, eligibility category, rural residence, managed care organization region, number of inpatient episodes in prior 6 months, number of outpatient episodes in prior 6 months, number of opioids fills in prior 6 months, number of benzodiazepine fills in prior 6 months, number of unique prescribers of opioids in prior 6 months, hepatitis C virus, HIV, anxiety disorder, mood disorder, schizophrenia and other psychosis, posttraumatic stress disorder, abscess, osteomyelitis, endocarditis, and soft skin tissue infection.

In the sensitivity analysis using the matched hospital sample, the DD estimate was slightly larger in magnitude than our main DD results (Table 3). We found that patients with index OUD ED visits in 2020 at O-HQIP–attesting hospitals were 3.6 percentage points more likely than patients treated at non–O-HQIP–attesting hospitals to receive buprenorphine treatment within 30 days (β, 0.036; 95% CI, 0.001-0.070). Results from the false treatment implementation date analysis of the parallel trends assumption (eTable 5 in Supplement 1) did not show a significant association between O-HQIP and 30-day buprenorphine treatment before O-HQIP implementation.

**Table 3.** Robustness Test of the Association of the Opioid Hospital Quality Improvement Program (O-HQIP) With Patient Treatment With Buprenorphine Within 30 Days of Index Emergency Department Visit, With Matched Hospital Sample (5052 Observations)^a^

Model	β (95% CI)		
	Overall before to after O-HQIP vs no O-HQIP implementation^b^	Year-specific indicators for post–O-HQIP period^b^	
		2019 O-HQIP attesting	2020 O-HQIP attesting
A: Unadjusted models			
Buprenorphine within 30 d	0.023 (−0.010 to 0.057)	0.016 (−0.027 to 0.058)	0.034 (0.000 to 0.068)^c^
B: Adjusted models			
Buprenorphine within 30 d	0.024 (−0.010 to 0.058)	0.015 (−0.029 to 0.060)	0.036 (0.001 to 0.070)^c^

^a^ In the model that evaluates the overall association, the coefficient of interest (O-HQIP attesting) is an indicator for whether a patient’s index opioid use disorder emergency department visit occurred during or after January 2019 (the start date for the O-HQIP) at O-HQIP–attesting hospitals. In the model that evaluates the year-specific associations, the 2 coefficients of interest are 2 indicator variables: 1 for patients visiting attesting hospitals in 2019 and 1 for 2020. All models included hospital-level fixed effects to control for time-invariant hospital unobserved effects. Adjusted regression models controlled patient characteristics, including age (at the time of index opioid use disorder visit), sex, race and ethnicity, eligibility category, rural residence, managed care organization region, number of inpatient episodes in prior 6 months, number of outpatient episodes in prior 6 months, number of opioids fills in prior 6 months, number of benzodiazepine fills in prior 6 months, number of unique opioid prescribers in prior 6 months, hepatitis C virus, HIV, anxiety disorder, mood disorder, schizophrenia and other psychosis, posttraumatic stress disorder, abscess, osteomyelitis, endocarditis, and soft skin tissue infection. The sample sizes of the overall and year-specific models are equal (ie, 5052). Clustering was performed at the hospital level.

^b^ No. (%) in the O-HQIP–attesting group before attestation, 64 (4.1%).

^c^ Statistically different from 0 at the 1%, 5%, and 10% level.

Table 4 reports the results from estimating the association between O-HQIP attestation and additional measures of SUD treatment. We did not find significant evidence of an association between O-HQIP–attesting hospitals and patients’ receipt of naltrexone, methadone, non-MOUD, or any SUD treatment within 30 days of their index OUD ED visit. However, the increase over time (2017-2018 vs 2019-2020) in the proportion of patients with a filed claim for any SUD treatment within 7 days of discharge in O-HQIP–attesting hospitals was 4.3 percentage points greater than we would have expected had the hospital not attested to the pathway (β, 0.043; 95% CI, 0.014-0.071).

**Table 4.** Association of the Opioid Hospital Quality Improvement Program (O-HQIP) With Patient Treatment With Additional Measures of Substance Use Disorder (SUD) Treatment (17 428 Observations)^a^

Outcome	β (95% CI)		
	Overall before to after O-HQIP vs no O-HQIP implementation	Year-specific indicators for post–O-HQIP period	
		2019 O-HQIP attesting	2020 O-HQIP attesting
Unadjusted models			
Naltrexone within 30 d	−0.003 (−0.014 to 0.007)	−0.007 (−0.023 to 0.010)	0.001 (−0.015 to 0.017)
Methadone within 30 d	−0.002 (−0.011 to 0.007)	−0.006 (−0.019 to 0.006)	0.003 (−0.006 to 0.013)
Non-MOUD within 30 d	0.007 (−0.032 to 0.046)	−0.012 (−0.058 to 0.044)	0.026 (−0.016 to 0.068)
SUD treatment within 30 d	0.018 (−0.020 to 0.055)	0.004 (−0.043 to 0.061)	0.029 (−0.015 to 0.072)
SUD treatment within 7 d	0.044 (0.015 to 0.073)^b^	0.016 (−0.016 to 0.053)	0.077 (0.039 to 0.115)^b^
Adjusted models			
Naltrexone within 30 d	−0.004 (−0.015 to 0.007)	−0.007 (−0.023 to 0.009)	0.000 (−0.015 to 0.015)
Methadone within 30 d	−0.002 (−0.011 to 0.007)	−0.007 (−0.019 to 0.005)	0.004 (−0.006 to 0.013)
Non-MOUD within 30 d	0.004 (−0.034 to 0.041)	−0.007 (−0.060 to 0.037)	0.024 (−0.016 to 0.063)
SUD treatment within 30 d	0.014 (−0.023 to 0.051)	0.009 (−0.045 to 0.054)	0.026 (−0.015 to 0.068)
SUD treatment within 7 d	0.043 (0.014 to 0.071)^b^	0.019 (−0.018 to 0.051)	0.077 (0.040 to 0.113)^b^

Abbreviation: MOUD, medication for opioid use disorder.

^a^ In the model that evaluates the year-specific associations, the 2 coefficients of interest are 2 indicator variables: 1 for patients visiting attesting hospitals in 2019 and 1 for 2020. All analyses used linear regression at the patient level and included an indicator variable for whether the patient was treated at an O-HQIP–attesting hospital after January 1, 2019. All models also included hospital-level fixed effects to control for time-invariant hospital unobserved effects. The sample sizes of the overall and year-specific models are equal (ie, 17 428). Clustering was performed at the hospital level.

^b^ Statistically different from 0 at the 5% level. Adjusted regression models also controlled patient characteristics, including age (at the time of index opioid use disorder emergency department visit), sex, race and ethnicity, eligibility category, rural residence, managed care organization region, number of inpatient episodes in prior 6 months, number of outpatient episodes in prior 6 months, number of opioids fills in prior 6 months, number of benzodiazepine fills in prior 6 months, number of unique opioid prescribers in prior 6 months, hepatitis C virus, HIV, anxiety disorder, mood disorder, schizophrenia and other psychosis, posttraumatic stress disorder, abscess, osteomyelitis, endocarditis, and soft skin tissue infection. In the model that evaluated the overall effect, the coefficient of interest (ie, O-HQIP attesting) is an indicator for whether a patient’s index opioid use disorder emergency department visit occurred during or after January 2019 (the start date for the O-HQIP) at O-HQIP–attesting hospitals.

## Discussion

In this cohort study, we found that a state program offering financial incentives to hospitals to develop OUD clinical pathways was associated with a 50% improvement in the rate of prescription fills for buprenorphine within 30 days of ED discharge. The program was associated with improvement in obtaining any SUD treatment within 7 days of discharge from the ED; however, there were no significant differences in receipt of any SUD treatment at 30 days. With surging rates of opioid overdose deaths,^22^ ED encounters present a crucial opportunity to engage patients with OUD treatment. Given high mortality rates immediately after ED discharge,^23,24^ treatment linkage should be timely and prioritize connection to facilities and clinicians able to initiate or continue medication treatment, the most effective modality for treating OUD. The O-HQIP may be a new policy approach to expanding access to evidence-based treatment for OUD across a diverse and large population of hospitals.

Recognition that MOUD should be initiated in the ED setting began after a landmark randomized controlled trial found that patients who received buprenorphine in the ED, compared with those who only received a referral to treatment, had significantly higher retention in addiction treatment and lower opioid misuse at 30 days after discharge.^16^ Despite this and other evidence supporting ED-initiated buprenorphine, patients with OUD infrequently receive MOUD, including buprenorphine, during an ED encounter. A recent study found that less than 10% of all patients in the US were prescribed buprenorphine within 30 days of their ED visit for an opioid overdose.^25^ The baseline rate for patients included in our study was even lower at approximately 5%.

Barriers to ED-initiated MOUD have been described for clinicians (eg, competing demands, lack of training and protocols),^26,27^ patients (eg, stigma, low readiness to initiate treatment, social factors),^28,29,30,31^ and health systems (eg, lack of personnel, weak referral relationships between hospitals and outpatient care).^27,32^ To address clinician and health system barriers, hospitals have attempted various implementation strategies for ED-initiated buprenorphine. However, only a few states have sought to implement OUD pathways across multiple hospitals and health systems. Legislation in Massachusetts and Rhode Island mandates that EDs offer specialized OUD evaluations and arrange linkage to treatment.^33,34,35^ Another initiative is the CA Bridge program, which selected 52 hospitals in California to participate.^36^ This program provided hospitals with funding to support clinician champions and substance use navigators and technical assistance in the form of implementation facilitation, clinician training, and treatment guidelines.

Pennsylvania’s O-HQIP program is novel in that all hospitals were eligible to participate and receive financial incentives. The first phase of this incentive program was designed to develop the infrastructure, resources, and expertise needed to deliver evidence-based treatment for ED patients with OUD. In the second and ongoing phase, hospitals are accountable for sustaining and improving the rates at which ED patients obtain follow-up addiction treatment. Future work is needed to evaluate whether patient outcomes continue to improve; explore treatment initiation after 30 days; evaluate the role of ED readmissions; and identify additional incentives or resources needed to optimize this important care transition at federal, state, and local levels.

### Limitations

This study has several limitations. First, our study focused only on a single state’s program and measured its potential effectiveness within the state’s Medicaid population. With respect to study generalizability, our findings might not reflect non-Medicaid populations or Medicaid populations in other states. However, pathways to improve care coordination and implement buprenorphine may benefit all patients with ED visits for opioid-related illnesses. Second, because the O-HQIP did not specify protocols and standards for participants, the program’s implementation processes across hospitals may have been heterogeneous in ways we could not observe. Future research should focus on understanding the implementation processes and mechanisms associated with patient outcomes. Third, given the absence of penalties for not meeting follow-up metrics, it is unclear how many hospitals successfully implemented their attested pathway.^10^ Fourth, this study only compared patients seen in hospitals that attested to adopting the pathway of prescribing buprenorphine in the ED with patients seen in hospitals that did not attest to any pathway. However, regardless of their attestation, all hospitals were eligible to receive incentive payments in 2020 if they achieved Pennsylvania DHS’s prespecified performance improvement targets for postdischarge SUD treatment. Our analysis can only estimate the outcomes associated with the initial attestation and cannot assess those of subsequent performance metric–based incentives because we lacked a control group to study those incentives. Studying the universal incentives would require comparison with Medicaid patients in another state that did not have a similar incentive program. Fifth, because OUD diagnosis codes in claims have limited sensitivity and specificity, our study may have inaccurately estimated the number of patients with OUD. However, we applied codes commonly used in related literature.^37,38,39^ Sixth, our DD analysis assumes that there are no unmeasured characteristics that differed between attesting and nonattesting hospitals associated with changes over time in the incidence of buprenorphine treatment. If such an unmeasured confounder exists, our estimates would be biased. Seventh, our study only considered the first OUD ED encounter for patients who had multiple encounters between 2017 and 2020.

## Conclusions

In this cohort study with a DD analysis of Medicaid adult enrollees treated in hospitals attesting or not attesting to an ED buprenorphine treatment pathway, we found that participation in the O-HQIP was associated with increases in patients obtaining buprenorphine treatment within 30 days of an OUD ED encounter. State programs that use financial incentives to drive OUD treatment practice changes in hospitals may be effective in improving quality and care transitions. Similar programs across the nation should be considered as part of a multifaceted approach to mitigating the opioid epidemic.

## Notes

Supplement 1. **eTable 1.** Codes Used to Identify Opioid Use Disorder (OUD) Emergency Department (ED) Encounters and Medication for OUD **eTable 2.** List of Hospital-Level and Hospital-Level Patient Composition Characteristics That May Be Associated With Patients’ Probability of Continuing Buprenorphine Treatment **eTable 3.** Characteristics of the Study Population, With Matched Hospital Sample **eTable 4.** Covariate Estimates From Unadjusted Analyses using the Main Model Specification **eTable 5.** Association of the Opioid Hospital Quality Improvement Program (O-HQIP) Attesting to Patient Treatment With Buprenorphine Within 30 Days of Index Emergency Department (ED) Visit, With April 2018 as the Implementation Date and Preintervention Data **eFigure.** Exploratory Plot Showing the Unadjusted Monthly Prevalence of 30-Day Buprenorphine Treatment for Patients Who Visited Opioid Hospital Quality Improvement Program (HQIP)– and non–Opioid (HQIP)–Attesting Hospitals, 2017-2020

Supplement 2.
Data Sharing Statement
