# Supplementary material for: Association Between Hospital Adoption of an Emergency Department Treatment Pathway for Opioid Use Disorder and Patient Initiation of Buprenorphine After Discharge
Source: JAMA Health Forum. 2023 Mar 24;4(3):e230245. doi: 10.1001/jamahealthforum.2023.0245 (PMC10313142; doi:10.1001/jamahealthforum.2023.0245)
Supplement: Supplement 1. — eTable 1. Codes Used to Identify Opioid Use Disorder (OUD) Emergency Department (ED) Encounters and Medication for OUD eTable 2. List of Hospital-Level and Hospital-Level Patient Composition Characteristics That May Be Associated With Patients’ Probability of Continuing Buprenorphine Treatment eTable 3. Characteristics of the Study Population, With Matched Hospital Sample eTable 4. Covariate Estimates From Unadjusted Analyses using the Main Model Specification eTable 5. Association of the Opioid Hospital Quality Improvement Program (O-HQIP) Attesting to Patient Treatment With Buprenorphine Within 30 Days of Index Emergency Department (ED) Visit, With April 2018 as the Implementation Date and Preintervention Data eFigure. Exploratory Plot Showing the Unadjusted Monthly Prevalence of 30-Day Buprenorphine Treatment for Patients Who Visited Opioid Hospital Quality Improvement Program (HQIP)– and non–Opioid (HQIP)–Attesting Hospitals, 2017-2020 [file jamahealthforum-e230245-s001.pdf]

Supplemental Online Content

Solomon KT, O'Connor J, Gibbons JB, et al. Association between hospital adoption of an emergency department treatment pathway for opioid use disorder and patient initiation of buprenorphine after discharge. *JAMA Health Forum*. 2023;4(3):e230245. doi:10.1001/jamahealthforum.2023.0245

eTable 1. Codes Used to Identify Opioid Use Disorder (OUD) Emergency Department (ED) Encounters and Medication for OUD

eTable 2. List of Hospital-Level and Hospital-Level Patient Composition Characteristics That May Be Associated With Patients' Probability of Continuing Buprenorphine Treatment

eTable 3. Characteristics of the Study Population, With Matched Hospital Sample

eTable 4. Covariate Estimates From Unadjusted Analyses using the Main Model Specification

eTable 5. Association of the Opioid Hospital Quality Improvement Program (O-HQIP) Attesting to Patient Treatment With Buprenorphine Within 30 Days of Index Emergency Department (ED) Visit, With April 2018 as the Implementation Date and Preintervention Data

eFigure. Exploratory Plot Showing the Unadjusted Monthly Prevalence of 30-Day Buprenorphine Treatment for Patients Who Visited Opioid Hospital Quality Improvement Program (HQIP)– and non–Opioid (HQIP)–Attesting Hospitals, 2017-2020

This supplemental material has been provided by the authors to give readers additional information about their work.

© 2023 Solomon KT et al. *JAMA Health Forum.*

eTable 1. Codes Used to Identify Opioid Use Disorder (OUD) Emergency Department (ED) Encounters and Medication for OUD

NDC codes for buprenorphine	00054017613, 00054017713, 00054018813, 00054018913, 00093537856, 00093537956, 00093572056, 00093572156, 00228315303, 00228315373, 00228315403, 00228315473, 00228315503, 00228315567, 00228315573, 00228315603, 00228315673, 00378092393, 00378092493, 00378876716, 00378876793, 00378876816, 00378876893, 00406192303, 00406192309, 00406192403, 00406192409, 00406800503, 00406802003, 00490005100, 00490005130, 00490005160, 00490005190, 00517072505, 00781721606, 00781721664, 00781722706, 00781722764, 00781723806, 00781723864, 00781724906, 00781724964, 12496010001, 12496010005, 12496030001, 12496030005, 12496120201, 12496120203, 12496120401, 12496120403, 12496120801, 12496120803, 12496121201, 12496121203, 12496127802, 12496128302, 12496130602, 12496131002, 16590066605, 16590066630, 16590066705, 16590066730, 16590066790, 23490927003, 23490927006, 23490927009, 35356000407, 35356000430, 35356055530, 35356055630, 42023017910, 42291017430, 42291017530, 42858050103, 42858050203, 43063018407, 43063018430, 43063066706, 43063075306, 43598057901, 43598057930, 43598058001, 43598058030, 43598058101, 43598058130, 43598058201, 43598058230, 47781035503, 47781035603, 47781035703, 47781035803, 49349055402, 49999039507, 49999039515, 49999039530, 49999063830, 49999063930, 50090157100, 50090292400, 50268014411, 50268014415, 50268014511, 50268014515, 50383028793, 50383029493, 50383092493, 50383093093, 52125067802, 52427069203, 52427069211, 52427069403, 52427069411, 52427069803, 52427069811, 52427071203, 52427071211, 52440010014, 52959030430, 52959074930, 53217013830, 53217024630, 54123011430, 54123090730, 54123091430, 54123092930, 54123095730, 54123098630, 54569549600, 54569573900, 54569573901, 54569573902, 54569639900, 54569640800, 54569657800, 54868570700, 54868570701, 54868570702, 54868570703, 54868570704, 54868575000, 55045378403, 55154496204, 55700014730, 55700018430, 55700030230, 55700030330, 55887031204, 55887031215, 58284010014, 59385001201, 59385001230, 59385001401, 59385001430, 59385001601, 59385001630, 60429058630, 60429058633, 60429058730, 60429058733, 61786067802, 61786091102, 61786091202, 62175045232, 62175045832, 62756045983, 62756046083, 62756096983, 62756097083, 63629402801, 63629402802, 63629403401, 63629403402, 63629403403, 63629403404, 63629409201, 63629409202, 63629507401, 63874108403, 63874108503, 63874117303, 63874117403, 64725093004, 64725192404, 65162041503, 65162041509, 65162041603, 65162041609, 66336001530, 66336001630, 68071138003, 68071151003, 68258299103, 68258299903, 68308020230, 68308020830
ED procedure codes for diagnoses of opioid dependence, withdrawal, abuse, and overdose	99281, 99282, 99283, 99284, 99285, G0380, G0381, G0382, G0383, G0384, G8354, G0385
ED revenue codes for diagnoses of opioid dependence, withdrawal, abuse, and overdose	0450, 0451, 0452, 0456, 0459, 0981
All professional and outpatient claims with a procedure code for buprenorphine or buprenorphine/naloxone	J0571, J0572, J0573, J0574, J0575
ICD-10 "F11" codes for OUD diagnoses	F1110, F1111, F11120, F11121, F11122, F11129, F1114, F11150, F11151, F11159, F11181, F11182, F11188, F1119, F1120, F1121, F11220, F11221, F11222, F11229, F1123, F1124, F11250, F11251, F11259, F11281, F11282, F11288, F1129, F1190, F11920, F11921, F11922, F11929, F1193, F1194, F11950, F11951, F11959, F11981, F11982, F11988, F1199
ICD-10 "T40" codes for opioid overdose diagnoses	T400X1A, T400X1D, T400X1S, T400X2A, T400X2D, T400X2S, T400X3A, T400X3D, T400X3S, T400X4A, T400X4D, T400X4S, T400X5A, T400X5D, T400X5S, T401X1A, T401X1D, T401X1S, T401X2A, T401X2D, T401X2S, T401X3A, T401X3D, T401X3S, T401X4A, T401X4D, T401X4S, T402X1A, T402X1D, T402X1S, T402X2A, T402X2D,

© 2023 Solomon KT et al. *JAMA Health Forum.*

	T402X2S, T402X3A, T402X3D, T402X3S, T402X4A, T402X4D, T402X4S, T402X5A, T402X5D, T402X5S, T403X1A, T403X1D, T403X1S, T403X2A, T403X2D, T403X2S, T403X3A, T403X3D, T403X3S, T403X4A, T403X4D, T403X4S, T403X5A, T403X5D, T403X5S, T404X1A, T404X1D, T404X1S, T404X2A, T404X2D, T404X2S, T404X3A, T404X3D, T404X3S, T404X4A, T404X4D, T404X4S, T404X5A, T404X5D, T404X5S, T40601A, T40601D, T40601S, T40602A, T40602D, T40602S, T40603A, T40603D, T40603S, T40604A, T40604D, T40604S, T40605A, T40605D, T40605S, T40691A, T40691D, T40691S, T40692A, T40692D, T40692S, T40693A, T40693D, T40693S, T40694A, T40694D, T40694S, T40695A, T40695D, T40695S

© 2023 Solomon KT et al. *JAMA Health Forum.*

eTable 2. List of Hospital-Level and Hospital-Level Patient Composition Characteristics That May Be Associated With Patients' Probability of Continuing Buprenorphine Treatment

Hospital-level and hospital-level patient-composition characteristics that may affect patients' probability of continuing buprenorphine treatment	non-profit status, teaching hospital designation, system membership, critical access designation, number of licensed beds, number of total inpatient admissions, percentage of admissions where Medicaid was the primary payor, number of psychiatric beds, mean patient age, patient race and ethnicity proportions, the proportion of patients living in a rural residence, and the total count of patient Elixhauser comorbidities of study patients

Notes. The 2018 American Hospital Association data was used to identify hospital. Patient characteristics were obtained using the Medicaid inpatient, outpatient, and enrollment files from 2018.

© 2023 Solomon KT et al. *JAMA Health Forum.*

eTable 3. Characteristics of the Study Population, With Matched Hospital Sample

	**O-HQIP attesting**		**Non-O-HQIP attesting**	
Time period	**2017-2018**	**2019-2020**	**2017-2018**	**2019-2020**
	**n or mean (% or SD)**	**n or mean (% or SD)**	**n or mean (% or SD)**	**n or mean (% or SD)**
Treatment with Buprenorphine (n,%)				
Buprenorphine within 30 Days of Index Visit	64 (4.1)	79 (7.5)	84 (5.1)	47 (5.9)
Patient Characteristics				
Age at index visit (mean, SD)	36.71 (10.68)	37.96 (11.00)	35.61 (10.33)	37.69 (10.95)
Male (n, %)	803 (51.8)	585 (55.6)	867 (52.6)	435 (54.3)
**Race/ethnicity**				
Hispanic	80 (5.2)	71 (6.7)	92 (5.6)	54 (6.7)
Non-Hispanic Black	246 (15.9)	215 (20.4)	114 (6.9)	84 (10.5)
Non-Hispanic White	1184 (76.4)	733 (69.7)	1405 (85.2)	637 (79.5)
Other race/ethnic groups[a]	40 (2.6)	33 (3.1)	38 (2.3)	26 (3.2)
**Eligibility Category (n, %)**				
Children	44 (2.8)	18 (1.7)	55 (3.3)	22 (2.7)
Disabled or Chron. Ill	265 (17.1)	189 (18.0)	239 (14.5)	147 (18.4)
Non-Disabled Adults	249 (16.1)	174 (16.5)	228 (13.8)	129 (16.1)
Expansion Adults	961 (62.0)	661 (62.8)	1083 (65.7)	495 (61.8)
**Managed Care Organization Region (n, %)**				
Lehigh Capital	337 (21.7)	223 (21.2)	260 (15.8)	122 (15.2)
New East	378 (24.4)	214 (20.3)	928 (56.3)	428 (53.4)
New West	67 (4.3)	37 (3.5)	113 (6.9)	72 (9.0)
Southeast	541 (34.9)	449 (42.7)	225 (13.6)	125 (15.6)
Southwest	227 (14.6)	129 (12.3)	123 (7.5)	54 (6.7)
**Address in rural area (n, %)**	198 (12.8)	129 (12.3)	306 (18.6)	207 (25.8)
**Comorbidities (n, %)**				
Hepatitis C virus	637 (41.1)	445 (42.3)	677 (41.1)	345 (43.1)
Anxiety disorder	704 (45.4)	437 (41.5)	743 (45.1)	355 (44.3)
Mood disorder	107 (6.9)	99 (9.4)	157 (9.5)	69 (8.6)
Schizophrenia or other psychotic disorder	125 (8.1)	110 (10.5)	99 (6.0)	82 (10.2)
Posttraumatic stress disorder	658 (7.2)	510 (9.3)	119 (6.3)	99 (10.5)
Skin and soft tissue infections	1305 (14.3)	620 (11.3)	257 (13.5)	119 (12.6)
Hospital characteristics (n, SD)				
Number of outpatient visits	3.61 (5.74)	3.42 (4.66)	3.43 (4.42)	3.49 (4.42)
Number of inpatient admissions	0.43 (1.24)	0.41 (1.24)	0.34 (0.81)	0.34 (0.98)
Number of opioid prescriptions fills	0.98 (2.57)	0.42 (1.63)	0.88 (2.64)	0.47 (1.71)
Number of benzodiazepine prescription	0.83 (2.12)	0.52 (1.75)	0.85 (2.12)	0.65 (1.96)
Elixhauser comorbidity index score at the index ED visit	2.11 (2.51)	2.12 (2.59)	1.94 (2.36)	1.99 (2.53)
Number of unique providers prescribing opioids	206 (13.3)	92 (8.7)	231 (14.0)	96 (12.0)
N	1550	1052	1649	801

Notes. The number of outpatient visits, inpatient admissions, opioid prescriptions, benzodiazepine prescription fills, and unique providers prescribing opioids are measured six months prior to the patient's index ED visit. For confidentiality purposes and adherence to Centers for Medicare & Medicaid Services' regulations, we do not report statistics when a small number of individuals, i.e., less than 11 individuals, had the relevant characteristic.

[a] Other race/ethnic group includes American Indian, Alaska Native, Asian, Native Hawaiian, and Pacific Islander

© 2023 Solomon KT et al. *JAMA Health Forum.*

eTable 4. Covariate Estimates From Unadjusted Analyses using the Main Model Specification

Variable:	Coefficient	Confidence interval	
Buprenorphine within 30 days	0.025**	0.005	0.047
Age at index visit	0.000	-0.001	0.000
Male	0.013	0.006	0.020
Race/Ethnicity			
Hispanic	-0.002	-0.012	0.008
Non-Hispanic Black	-0.017	-0.028	-0.007
Other race/ethnicity [a]	0.000	-0.019	0.019
Eligibility Categories			
Pregnant Women			
Children	-0.060	-0.096	-0.023
Disabled or Chron. Ill	-0.055	-0.085	-0.025
Non-Disabled Adults	-0.034	-0.064	-0.004
Expansion	-0.042	-0.071	-0.012
Rural	-0.005	-0.022	0.012
MCO Regions			
Lehigh/Capital			
New East	0.012	-0.008	0.031
New West	0.000	-0.040	0.039
Southeast	-0.011	-0.033	0.012
Southwest	0.021	-0.011	0.052
Number of out-patient visits in 6 months prior to index admission	-0.001	-0.001	0.000
Number of in-patient admissions in 6 months prior to index admission	-0.002	-0.004	0.001
Number of benzodiazepine prescription fills in 6 months prior to index admission	-0.001	-0.002	0.001
Number of study patient opioid prescription fills in 6 months prior to index admission	-0.001	-0.003	0.001
Number of unique opioid prescribers in 6 months prior to index admission	0.002	-0.004	0.007
Comorbidities related to OUD			
Hepatitis C	0.010	-0.002	0.022
Hepatitis B	0.000	-0.049	0.048
HIV	-0.021	-0.039	-0.002
Anxiety Disorder	0.014	0.004	0.024
Mood disorder	-0.001	-0.008	0.006
Schizophrenia and other psychotic disorders	-0.012	-0.022	-0.001
PTSD	0.007	-0.008	0.022
Intracranial and Intraspinal Abscess	0.002	-0.071	0.076
Osteomyelitis	-0.008	-0.043	0.027
Endocarditis	0.010	-0.047	0.067
Soft Skin Tissue Infection	0.013	0.000	0.027

Notes. All analyses used linear regression at the patient level and included an indicator variable for if the patient was treated at an O-HQIP attesting hospital after January 1, 2019. All models also included hospital-level fixed effects to control for time-invariant hospital unobserved effects. The sample sizes of the overall and year-specific models are equal, i.e., 17428. Clustering was performed at the hospital level. ** = statistically different from zero at the 5% level. Adjusted regression models also controlled patient characteristics, including age (at the time of index OUD visit), gender, race/ethnicity, eligibility category, rural residence, Managed Care Organization region, number of inpatient episodes in prior six months, number of outpatient episodes in prior six months, number of opioids fills in prior six months, number of benzodiazepine fills in prior six months, number of unique opioid prescribers in prior six months, Hepatitis C virus, human immunodeficiency virus, anxiety disorder, mood disorder, schizophrenia and other psychosis, posttraumatic stress disorder, abscess, osteomyelitis, endocarditis, and soft skin tissue infection. In the model that examines the overall effect, the coefficient of interest, i.e., “O-HQIP attesting,” is an indicator for whether a patient’s index ED OUD visit occurred during or after January 2019 (the start date for the O-HQIP) at O-HQIP attesting hospital.

a Other race/ethnic group includes American Indian, Alaska Native, Asian, Native Hawaiian, and Pacific Islander

© 2023 Solomon KT et al. *JAMA Health Forum.*

eTable 5. Association of the Opioid Hospital Quality Improvement Program (O-HQIP) Attesting to Patient Treatment With Buprenorphine Within 30 Days of Index Emergency Department (ED) Visit, With April 2018 as the Implementation Date and Preintervention Data

**Model**	**Overall**		
Variable:	**O-HQIP attesting**		
Statistics:	Coefficient	95% CI	
Outcomes:			
**Panel A: Unadjusted Models**	0.011	-0.016	0.038
Buprenorphine within 30 Days			
**Panel B: Adjusted Models**			
Buprenorphine within 30 Days	0.009	-0.018	0.036
Observations	11019		

All analyses used linear regression at the patient level and included an indicator variable for if the patient was treated at an O-HQIP attesting hospital after April 1, 2018. All models also included hospital-level fixed effects to control for time-invariant hospital unobserved effects. Clustering was performed at the hospital level. ** = statistically different from zero at the 5% level. Adjusted regression models also controlled patient characteristics, including age (at the time of index OUD visit), gender, race/ethnicity, eligibility category, rural residence, Managed Care Organization region, number of inpatient episodes in prior six months, number of outpatient episodes in prior six months, number of opioids fills in prior six months, number of benzodiazepine fills in prior six months, number of unique opioid prescribers in prior six months, Hepatitis C virus, human immunodeficiency virus, anxiety disorder, mood disorder, schizophrenia and other psychosis, posttraumatic stress disorder, abscess, osteomyelitis, endocarditis, and soft skin tissue infection.

© 2023 Solomon KT et al. *JAMA Health Forum.*

eFigure. Exploratory Plot Showing the Unadjusted Monthly Prevalence of 30-Day Buprenorphine Treatment for Patients Who Visited Opioid Hospital Quality Improvement Program (HQIP)– and non–Opioid (HQIP)–Attesting Hospitals, 2017-2020

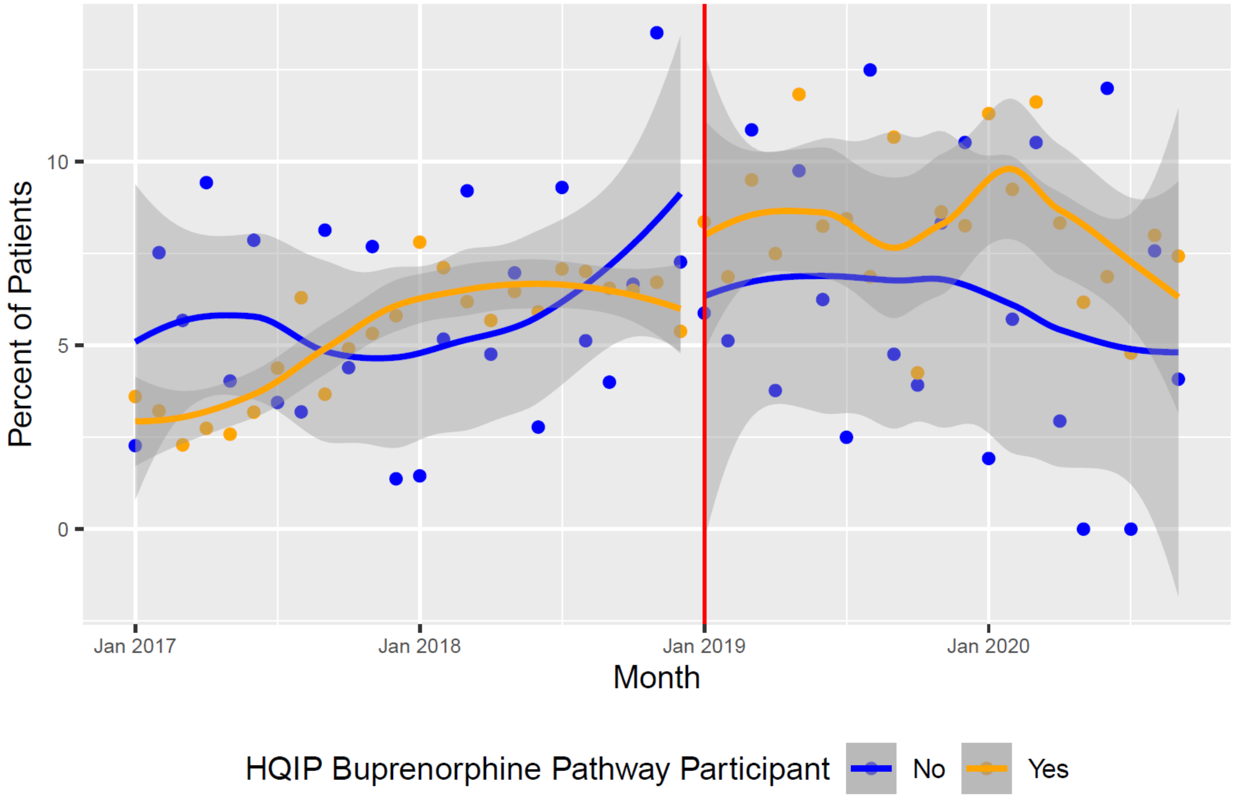

Notes. This figure shows the unadjusted monthly prevalence of 30-day buprenorphine treatment for Medicaid enrollees aged 18 to 64 years who were treated in hospitals attesting to an ED buprenorphine treatment O-HQIP pathway or non-O-HQIP attesting hospitals from 2017 to 2020. See Figure 1 for included study participants.

© 2023 Solomon KT et al. *JAMA Health Forum.*
