# Supplementary material for: Association Between Hospital Adoption of an Emergency Department Treatment Pathway for Opioid Use Disorder and Patient Initiation of Buprenorphine After Discharge
Source: JAMA Health Forum. 2023 Mar 24;4(3):e230245. doi: 10.1001/jamahealthforum.2023.0245 (PMC10313142; doi:10.1001/jamahealthforum.2023.0245)
Supplement: Supplement 2. — Data Sharing Statement [file jamahealthforum-e230245-s002.pdf]

Data Sharing Statement

Solomon. Association Between Hospital Adoption of an Emergency Department Treatment Pathway for Opioid Use Disorder and Patient Initiation of Buprenorphine After Discharge. *JAMA Health Forum*. Published March 24, 2023. doi:10.1001/jamahealthforum.2023.0245

Data
Data available: No

Additional Information
Explanation for why data not available: The authors used restricted access to Medicaid claims data. Researchers may contact the Pennsylvania Department of Human Services for data access.
